# Platycodin D enhances LDLR expression and LDL uptake via down-regulation of *IDOL* mRNA in hepatic cells

**DOI:** 10.1038/s41598-020-76224-w

**Published:** 2020-11-16

**Authors:** Yu-Jeong Choi, Sol Ji Lee, Hyo In Kim, Hee Jung Lee, So Jung Kang, Tai Young Kim, Chunhoo Cheon, Seong-Gyu Ko

**Affiliations:** 1 Department of Science in Korean Medicine, Graduate School, Kyung Hee University, Seoul, 02447 Republic of Korea; 2 Center for Cognition and Sociality, Institute for Basic Science, Daejeon, 34126 Republic of Korea; 3 Department Global Public Health and Korean Medicine Management, College of Korean Medicine, Graduate School, Kyung Hee University, Seoul, Republic of Korea; 4 Department of Clinical Koeran Medicine, Graduate School, Kyung Hee University, Seoul, Republic of Korea; 5 Department of Preventive Medicine, College of Korean Medicine, Kyung Hee University, 1 Hoegi, Seoul, 130-701 Korea

**Keywords:** Drug discovery, Molecular biology, Medical research, Molecular medicine

## Abstract

The root of *Platycodon grandiflorum* (PG) has long been used as a traditional herbal medicine in Asian country. Platycondin D (PD), triterpenoid saponin that is a main constituent of PG, exhibits various biological activities such as anti-inflammatory, anti-oxidant, anti-diabetic, and anti-cancer effects. A previous study showed that PD had cholesterol-lowering effects in mice that develop hypercholesterolemia, but the underlying molecular mechanisms have not been elucidated during the last decade. Here, we demonstrated that both PG and PD markedly increased levels of cell surface low-density lipoprotein receptor (LDLR) by down-regulation of the E3 ubiquitin ligase named inducible degrader of the LDLR (IDOL) mRNA, leading to the enhanced uptake of LDL-derived cholesterol (LDL-C) in hepatic cells. Furthermore, cycloheximide chase analysis and in vivo ubiquitination assay revealed that PD increased the half-life of LDLR protein by reducing IDOL-mediated LDLR ubiquitination. Finally, we demonstrated that treatment of HepG2 cells with simvastatin in combination with PG and PD had synergistic effects on the improvement of LDLR expression and LDL-C uptake. Together, these results provide the first molecular evidence for anti-hypercholesterolemic activity of PD and suggest that PD alone or together with statin could be a potential therapeutic option in the treatment of atherosclerotic cardiovascular disease.

## Introduction

Cholesterol is a principle component of cell membranes and serves as a precursor for the synthesis of bile acids and steroid hormones^1,2^. Most of cholesterol in the body is made by the liver and delivered to other organs through forms of low-density lipoprotein (LDL) particles that package cholesterol. Because high levels of LDL-derived cholesterol (LDL-C) in plasma, referred to as hypercholesterolemia, is a significant risk factor for atherosclerotic cardiovascular disease (CVD)^3,4^, lowering serum LDL-C has traditionally been considered as a therapeutic strategy for treating this disease.

The liver removes LDL-C from the blood by LDL receptor (LDLR), a cell surface protein that binds to LDL particles and mediates their uptake into cells via endocytosis^5^. LDLR expression is tightly regulated in response to the availability of intracellular cholesterol via transcriptional and post-translational pathways. The transcription of LDLR gene is controlled by sterol regulatory element binding protein-2 (SREBP-2)^6,7^. When intracellular cholesterol levels are low, the SREBP-2 located on the endoplasmic reticulum (ER) membrane is transported to the Golgi where the transcription factor domain of SREBP-2 is cleaved by two Golgi proteases, namely Site-1 and Site-2 proteases (S1P and S2P) and then translocated to nucleus. The cleaved mature form of SREBP-2 activates target genes including 3-Hydroxy-3-Methyl-glutaryl-Coenzyme A Reductase (HMGCR) and LDLR^8^. In addition, it has been studied in some details that LDLR is also regulated by post-translational mechanisms. Proprotein convertase subtilisin/kexin type 9 (PCSK9) binds to the extracellular domain of LDLR on the cell surface and interferes with recycling of the LDLR back to the plasma membrane after endocytosis for its subsequent lysosomal degradation^9^. Inducible degrader of the LDLR (IDOL), an E3 ubiquitin ligase, is also known to trigger degradation of the LDLR by ubiquitination-mediated lysosomal degradation mechanism^9,10^. Accordingly, these regulatory pathways govern hepatic LDLR abundance and, therefore, drugs targeting one of these pathways have been developed for elevating LDLR levels and subsequent enhancing clearance of LDL particles from the blood, consequently for treatment of hypercholesterolemia^11,12^.

Statins are a class of medications used to reduce blood cholesterol and commonly prescribed for CVD including stroke^13^. Statins inhibit the activity of HMGCR, a rate limiting enzyme in cholesterol biosynthesis, leading to a reduction in cholesterol content in the liver^13^. In response to the decreased levels of intracellular cholesterol, proteolytic processing of SREBP2 is enhanced and the active SREBP2 moves to nucleus where it binds to the promoter of LDLR, thereby inducing LDLR expression and LDL-C uptake. Although statins have been proven to be effective drugs for treating patients with high levels of LDL-C, many patients treated with statins fail to achieve LDL-C target values and can develop resistance or intolerance to this drug^14^. Therefore, new cholesterol-lowering drugs, alternatives to statins, are needed. Recently, FDA-approved anti-PCSK9 monoclonal antibodies, such as alirocumab and evolocumab, that block PCSK9 activity have emerged as a valuable addition to treatment options for LDL-C lowering therapy^15^. These PCSK9 inhibitors reduce the degradation of LDLR and increase the clearance of serum LDL-C. In several clinical trials, it was demonstrated that the PCSK9 inhibitors can be used for patients who experienced adverse effects of statins, or in combination with statins to boost their benefit^15^. But, because they are relatively expensive than other cholesterol medications, cost-effectiveness of anti-PCSK9 antibodies became a main issue limiting their widespread use. To date, IDOL inhibition is also considered as LDL-C lowering strategy. Although effective IDOL-targeted therapies have yet to be developed, several recent studies have demonstrated that natural compounds including docosahexanoic acid and xantholhumol modulate hepatic LDLR abundance via suppression of IDOL expression^16,17^. In addition, a recent study identified a cyclic peptide that disrupts IDOL homodimerization as an IDOL inhibitor and showed that it increased LDLR levels in HepG2 cells^18^.

Platycodin D (PD), a triterpene saponin isolated from the *platycodon grandiflorum* (PG), has been shown to regulate numerous biological processes involved in apoptosis, inflammation, oxidative stress, and hepatotoxicity^19–21^. Particularly, a previous study demonstrated the cholesterol lowering effect of PD in mouse models of hypercholesterolemia^22^. However, no studies have been conducted that address how PD affects hepatic cholesterol metabolism. Thus, in this study, we aim to understand molecular mechanisms by which PD exerts its effects on hypocholesterolemic action by focusing on its effects on LDLR expression and LDL-C uptake in HepG2 cells.

## Materials and methods

### Cell culture and chemicals

HepG2, SNU-387, and Hep3B cell lines were obtained from the Korean Cell Line Bank and cultured at 37 °C in a humidified 5% CO_2_ atmosphere. HepG2 and SNU-387 cells were maintained in RPMI-1640 and Hep3B cells was maintained in DMEM, and both culture media were supplemented with 10% fetal bovine serum (FBS) and 100 U/mL penicillin–streptomycin. PG was offered by Hanpoong Pharm and Foods Company (Korea) and dissolved in distilled water at a concentration of 20 mg/mL. PD and T0901317 were purchased from Cayman (USA). Simvastatin, Bafilomycin A1 (Baf A1) and cycloheximide (CHX) were purchased from Sigma-Aldrich (USA).

### Cell viability assay

The HepG2 cells were seeded in 96-well plate and treated with indicated doses of PG and PD. After 24 h incubation, WST solution (Daeillab, Korea) was added to each well and further incubated for 2 h. Water-soluble formazan formed in medium was measured at 450 nm absorption by the ELISA reader (Molecular Devices, USA).

### Western blot analysis

Proteins extracted from the hepatic cells were separated on 10% SDS-PAGE and transferred to nitrocellulose membranes (Thermo Scientific, USA).The membranes were incubated for overnight at 4 °C with the following primary antibodies: anti-LDLR, -IDOL, -HMGCR, and –LXRα (Abcam, USA), anti-GAPDH (Cell signaling, USA), anti-ABCA1 and -ABCG1 (Novus Biologicals, USA), anti-SREBP1, -SREBP2 (Santa Cruz, USA), anti-HA (BioLegend, USA) and anti-PCSK9 (Proteintech, USA). The blots were then incubated with HRP-conjugated secondary antibody (Thermo Scientific, USA) for 1 h at room temperature and incubated in the Pierce ECL Western Blotting Substrate (Thermo Scientific, USA).

### Reverse transcription (RT)-PCR and real-time PCR

Total RNA was extracted for hepatic cells treated with PG or PD using R&A-BLUE Total RNA Extraction Kit (Intron Biotechnology, Korea). The complementary DNA (cDNA) was synthesized from total RNA using PrimeScript 1st strand cDNA Synthesis Kit (Takara Biotechnology, China). RT-PCR was performed with Maxime PCR PreMix Kit (Intron Biotechnology, Korea) and GAPDH was used as the internal control. Real-time PCR for mRNA quantification were performed using SensiFAST Probe Hi-ROX Kit (Bioline, USA). The mRNA expression was normalized to the GAPDH and calculated using the 2^−ΔΔCt^ method. The human primers were the following: LDLR (Forward) 5′-CAGATATCATCAACGAAGC-3′, (Reverse) 5′-CCTCTCACACCAGTTCACTCC-3′; IDOL (Forward) 5′-TTGTGGACCTCGTTTCAAGA-3′, (Reverse) 5′-GCTGCAGTTCATGCTGCT-3′; GAPDH for RT-PCR (Forward) 5′-CGTCTTCACCACCATGGAGA-3′, (Reverse) 5′-CGGCCATCACGCCACAGTTT-3′; GAPDH for real-time PCR (Forward) 5′-AATCCCATCACCATCTTCCA-3′, (Reverse) 5′-TGGACTCCACGACGTACTCA-3'.

### LDL uptake assay

HepG2 cells were treated with drugs for 24 h and the culture media was replaced with fresh media containing 5 μg/ml BODIPY-FL-LDL (Invitrogen, USA). After 1 h incubation, the cells were washed with PBS containing 0.3% bovine serum albumin (BSA) and fixed with 4% paraformaldehyde (PFA) for 10 min. Nuclei was stained with 1 μg/ml DAPI in 2% BSA solution for 1 min. The stained cells were imaged using a confocal microscopy (Carl Zeiss, Germany) and immunofluorescence intensity was quantified by Image J software.

### Cell surface LDLR analysis

The HepG2 cells were treated as indicated above for 24 h, harvested and then incubated with the anti-LDLR antibody (1:60 dilution in PBS containing 5% BSA), at 37 °C for 30 min. The cells were washed with 1% BSA in PBS and incubated with Alexa Fluor 488-conjugated goat anti-rabbit IgG (Thermo Scientific, USA) using a 1:250 dilution at 37 °C for 30 min. The cells were fixed with 0.5% PFA for 10 min, resuspended in PBS, and measured by flow cytometry (FACSCalibur, BD Biosciences, USA). Data were analyzed using CellQuest Pro software version 5.2 (BD Biosciences, USA).

### Luciferase reporter assay

Hepatic cells were co-transfected with pLDLR-Luc plasmid (a gift from Axel Nohturfft, Addgene #14940) and Renilla luciferase reporter plasmid (pRL-TK) using Lipofectamine 3000 (Thermo Scientific, USA). After 24 h, the transfected cells were treated with 2.5 μM PD or 250 μg/mL PG for additional 24 h. Cell lysates were collected and luciferase activities were measured using Fluoroskan FL Microplate Luminometer (Thermo Scientific, USA) and Dual-Luciferase Reporter Assay System (Promega, USA) according to the manufacturer’s instructions. Luciferase activities were normalized to the Renilla luciferase expression.

### In vivo ubiquitination assay

HepG2 cells were transfected with HA-ubiquitin using Lipofectamine 3000 reagent (Thermo Scientific, USA). After overnight incubation, the cells were treated or co-treated with 2.5 μM PD and 10 μM T0901317 for 18 h and added with 50 nM Baf A1 for 6 h prior to harvesting the cells. The cell pellets were freeze–thawed twice before resuspension in 100 μl SDS lysis buffer (50 mM Tris–HCl, pH 7.5, 0.5 mM EDTA, 1% SDS and protease inhibitor) and boiled for 10 min. The boiled lysates were diluted with 900 μl NP-40 buffer (25 mM Tris–HCl, pH 7.5, 150 mM NaCl, 0.5% NP-40) and rotated for 10 min at cold room. Following centrifugation at 13,000 rpm for 10 min at 4 °C, 25 μl of lysate was used for input sample and the remaining lysate was immunoprecipitated with anti-LDLR overnight at 4 °C, followed by further incubation with protein A/G agarose (Thermo Scientific, USA) for 1 h at 4 ℃. The beads were washed three times with 1 ml of lysis buffer, re-suspended in 2 × SDS sample buffer, boiled at 95℃, and then separated by SDS-PAGE.

### Statistical analysis

Data is presented as the mean ± standard deviation (SD). Statistical analysis was performed using student’s *t*-test or one-way ANOVA followed by Tukey’s post hoc test. *p *values < 0.05 were considered to represent the significant difference.

## Results

### Cytotoxic effects of PG and PD on HepG2 cells

**Figure. 1:**
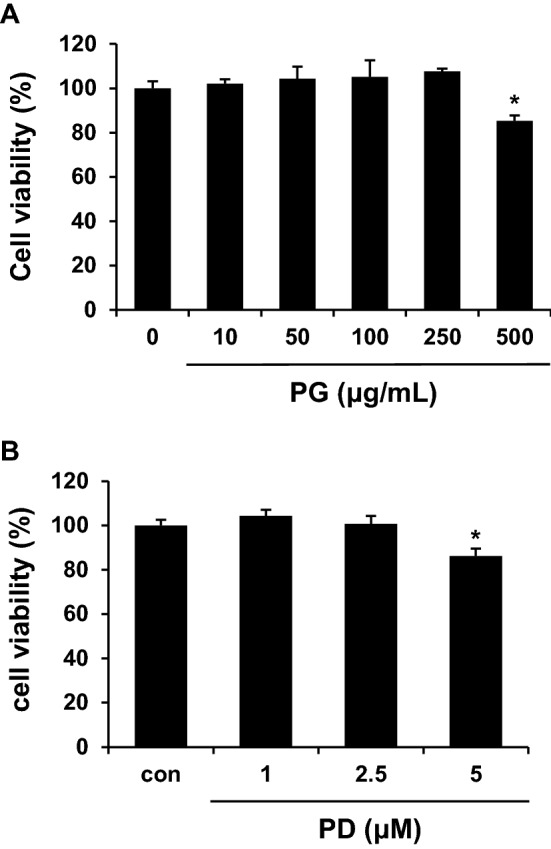
Effects of PG and PD on the viability of HepG2 cells. HepG2 cells were treated with indicated concentrations of PG (**A**) and PD (**B**) for 24 h. Cell viability was measured using a WST-8. Data represented as mean ± standard deviation (SD). *P < 0.05 by Student’s *t* tests.

To investigate whether PG and PD display toxic effects on HepG2 cells, cell viability was determined by the WST-8 assay following treatment with different concentrations of PG (10, 50, 100, 250, 500 μg/mL) and PD (1, 2.5, 5 μM) for 24 h. The results showed that 10–250 μg/mL of PG and 1–2.5 μM of PD have no significant cytotoxic effects on HepG2 cells, but 500 μg/mL PG and 5 μM PD reduce cell viability around 15% compared to control (Fig. 1A,B).

### PG and PD induce cell surface LDLR expression and LDL-C uptake in HepG2 cells

**Figure. 2:**
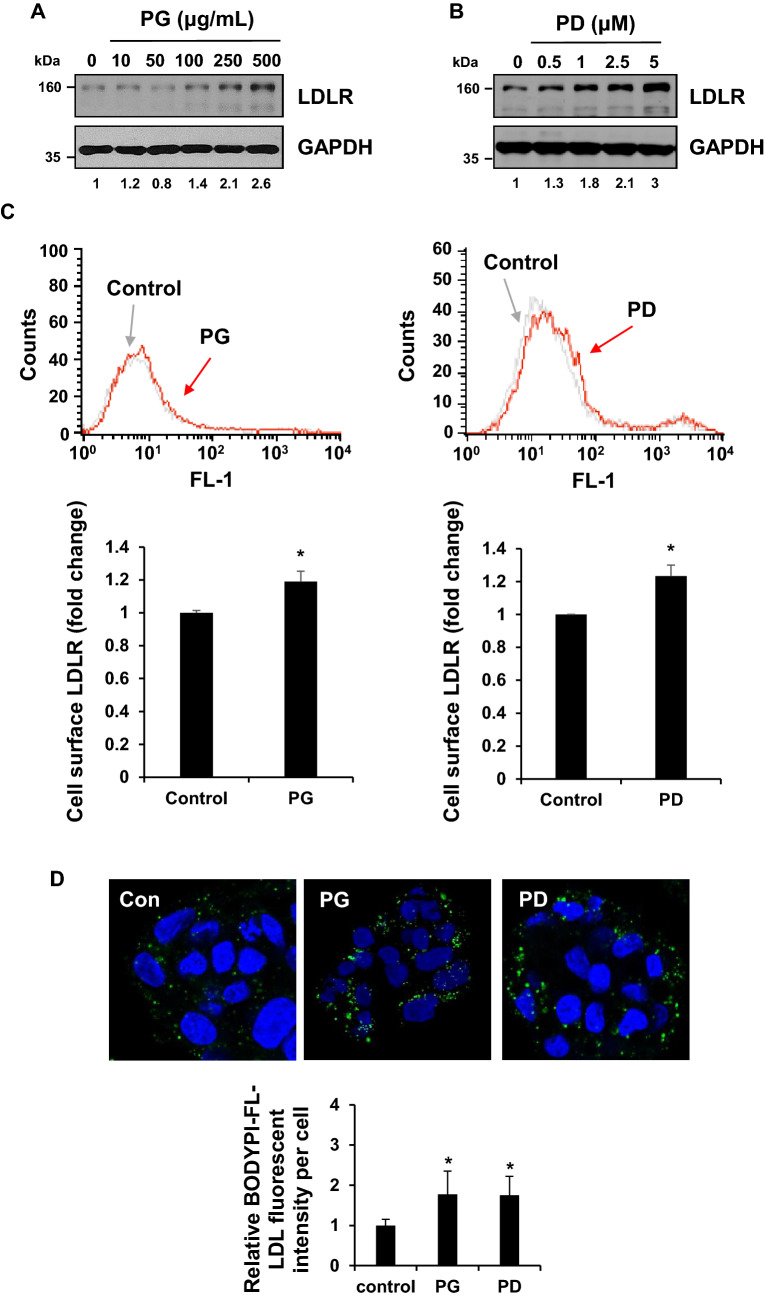
PG and PD induce LDLR expression and LDL-C uptake in HepG2 cells. (**A**, **B**) HepG2 cells were treated with indicated concentration of PG or PD for 24 h. Cell lysates were subjected to western blotting with anti-LDLR and anti-GAPDH antibodies. (**C**) HepG2 cells were treated with 250 μg/ml PG and 2.5 μM PD for 24 h, followed flow cytometry to determine the amount of cell surface LDLR expression. Data were analyzed using CellQuest Pro software version 5.2 and the average fluorescence intensity of LDLR was shown as fold change. Error bar represented the mean ± SD. *P < 0.05 by Student’s *t* tests. (**D**) HepG2 cells were treated with 250 μg/ml PG and 2.5 μM PD for 24 h, followed by incubation with 5 μg/mL Bodipy FL dye-labeled LDL for 1 h. The internalization of the fluorescence labeled LDL (green) was imaged using confocal microscopy. DAPI (blue) was used for nuclear DNA staining. Quantification of LDL fluorescence intensity per cells was analyzed using Image J. Bar graph represents the mean ± SD. *P < 0.05 by one-way ANOVA with Tukey’s post hoc test.

LDLR is a cell surface receptor that mediates the uptake of LDL-C from plasma, lowering blood cholesterol level^23^. A previous study reported that PD has a cholesterol-lowing effect in mouse model which develop hypercholesterolemia^22^. Thus, we first explored whether PG and PD upregulate LDLR expression in HepG2 cells. Western blot analysis showed that the level of LDLR expression was stimulated from 100 μg/ml of PG and 0.5 μM of PD and further increased in a dose-dependent manner, reaching maximum induction of 2.6 and 3 folds relative to controls at 500 μg/ml of PG and 5 μM of PD, respectively (Fig. 2A,B). For the next experiments, we choose to use the concentration of 250 μg/ml PG and 2.5 μM PD, since cytotoxic effect was not exhibited at this concentration (Fig. 1). Next, we examined whether PG and PD increase LDLR expression on cell surface. FACS analysis revealed that cell surface level of LDLR was enhanced by 1.2 ± 0.06 and 1.2 ± 0.07 compared to control upon treatment of PG and PD, respectively (Fig. 2C). Finally, to investigate the effect of PG and PD on the uptake of LDL-C, we incubated PG or PD-treated HepG2 cells with BODYPI-labeled LDL particles for 1 h. The uptake of LDL particles was visualized by confocal microscopy and quantified by measuring the fluorescent intensity per cells. As shown in Fig. 2D, treatment of PG and PD caused a similar 1.7-fold increase in the uptake of LDL particles in HepG2 cells. Taken together, these results demonstrate that PG and its derived compound, PD lead to the induction of LDLR cell surface expression, thereby enhancing the uptake of LDL-C in HepG2 cells.

### PG and PD reduce *IDOL* mRNA expression in hepatic cells

**Figure 3:**
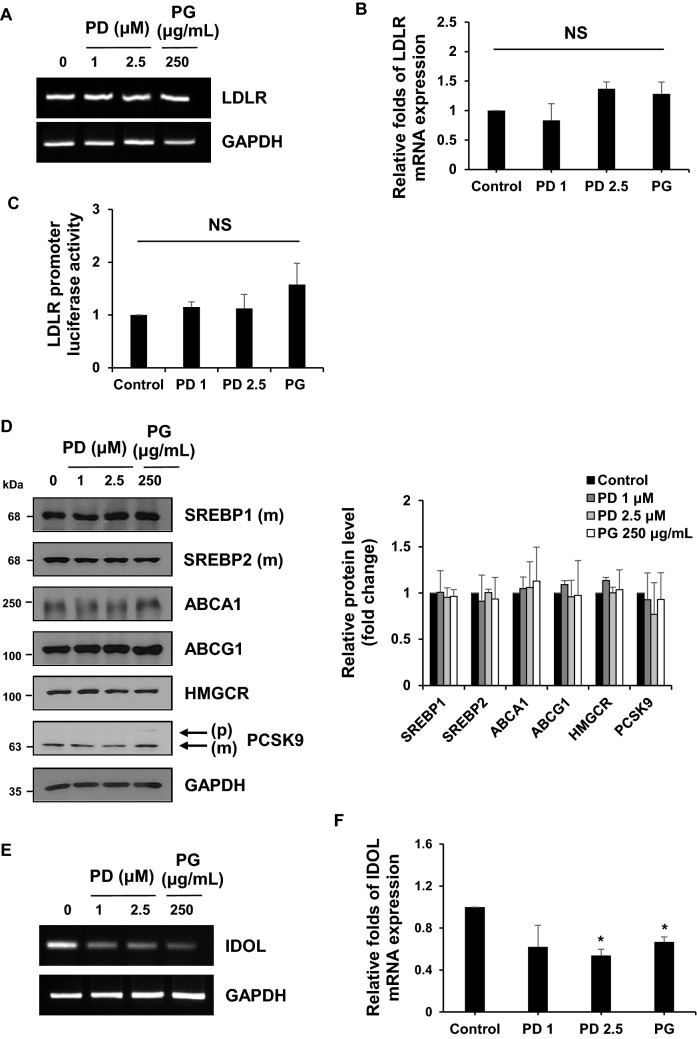
PG and PD inhibit *IDOL* transcription but not *LDLR* transcription and promoter activity in HepG2 cells. (**A**, **B**, **E**, **F**) HepG2 cells were treated with 250 μg/mL PG and 1, 2.5 μM PD for 24 h. RT-PCR and Real-time PCR assay were performed to measure the expression of *LDLR* mRNA (**A**, **B**) or *IDOL* mRNA (**E**, **F**). *GAPDH* was used as a reference gene for quantification analysis. Quantitative real-time PCR represent the mean ± SD from three independent experiments. *P < 0.05 by one-way ANOVA with Tukey’s post hoc test. *NS* not significant. (**C**) HepG2 cells were cotransfected with the pRL-TK vector and pLDLR-Luc plasmid. The cells were re-seeded in 12-well plate and treated with PG (250 μg/mL) or PD (1, 2.5 μM) for 24 h. The luciferase activities were measured and normalized with the respective Renilla activity. The data represent the mean ± SD of independent experiments. *NS* not significant. (**D**) After treatment of HepG2 cells with 250 μg/mL PG and 1, 2.5 μM PD for 24 h, cell lysates were subjected to western blotting with the indicated antibodies. The figure shows a representative western blot. The intensity of each protein bands from western blotting were determined by Image J software and normalized to that of GAPDH control. Bar graph shows the mean ± SD from three independent experiments.

**Figure 4:**
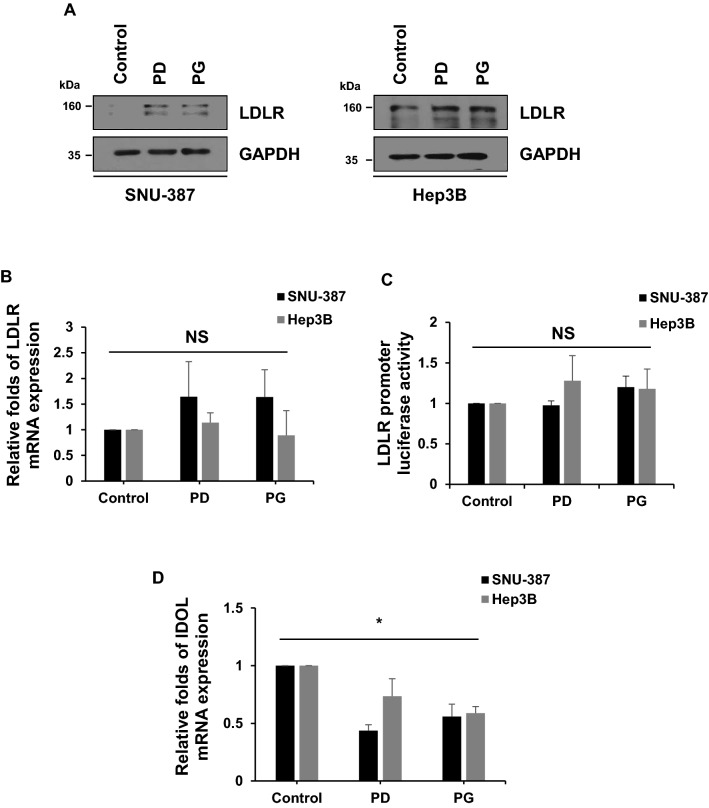
PG and PD increase LDLR protein levels by inactivation of *IDOL* mRNA in hepatic cell lines. (**A**) SNU-387 and Hep3B cells were treated with 2.5 μM PD and 250 μg/mL PG for 24 h. Cell lysates were analyzed by western blotting with anti-LDLR and anti-GAPDH antibodies. (**C**) Hepatic cells were co-transfected with and pLDLR-Luc and pRL-TK vector for 24 h followed by treatment with 2.5 μM PD and 250 μg/mL PG for 24 h. Luciferase activity was measured and normalized by Renilla luciferase expression. (**B**, **D**) Hepatic cells were treated with 2.5 μM PD and 250 μg/mL PG for 24 h. *LDLR* (**B**) and *IDOL* (**D**) mRNA expression was analyzed by real-time PCR. The data represent mean ± SD of three independent experiments. *P < 0.05 by one-way ANOVA with Tukey’s post hoc test.

LDLR expression is tightly regulated at multiple steps to maintain cholesterol homeostasis in cells. Especially, *LDLR* gene transcription is controlled by SREBP-2, a transcription factor whose proteolytic activation is dependent on cellular cholesterol levels^24^. LDLR protein is also known to undergo lysosomal degradation by PCSK9 or IDOL^25^. To define the molecular mechanisms underlying the increased LDLR expression by PG and PD, we first determined the effects of PG and PD on *LDLR* mRNA expression by reverse transcription polymerase chain reaction (RT-PCR) and quantitative real-time PCR. Unexpectedly, *LDLR* mRNA was not changed by the treatment of PG and PD (Fig. 3A,B). Furthermore, we confirmed that PG and PD did not increase LDLR promoter activity by the luciferase reporter assay (Fig. 3C). Consistent with these results, the amount of mature form of SREBPs were not altered upon treatment of each drug (Fig. 3D). In addition, we could not observe any significant changes in other proteins involved in regulating cholesterol metabolism, such as HMGCR, an enzyme for cholesterol synthesis and ABCA1/ABCG1, membrane transporters mediating cholesterol efflux (Fig. 3D). Therefore, we reasoned that the elevated levels of LDLR by PG and PD might be due to a post-translational mechanism. To test this, we investigated whether PG and PD have an inhibitory effect on PCSK9 that binds to LDLR and direct it to lysosome for degradation and found that both drugs had little effect on precursor and mature form of PCSK9 (Fig. 3D). We then tested their effects on IDOL, an E3 ubiquitin ligase that targets LDLR for its lysosomal degradation. Remarkably, both PG and PD decreased *IDOL* mRNA levels, as determined by RT-PCR and quantitative real-time PCR (Fig. 3E,F). In addition, we examined the effects of PG and PD on LDLR and IDOL expression in another hepatic cell lines including SNU-387 and Hep3B. Similar to HepG2 cells, upon treatment with PG and PD, LDLR protein was upregulated without a statistically significant increase in its mRNA level and promoter activity in both cells (Fig. 4A–C). Importantly, real-time PCR revealed that *IDOL* gene was down-regulated after treatment with both drugs in these hepatic cells (Fig. 4D). Collectively, these results suggest that IDOL, but not other cholesterol regulatory proteins, is involve in the PG and PD-mediated upregulation of LDLR in hepatic cells.

### PD enhances LDLR stability by inhibiting LXR-IDOL pathway in HepG2 cells

**Figure 5:**
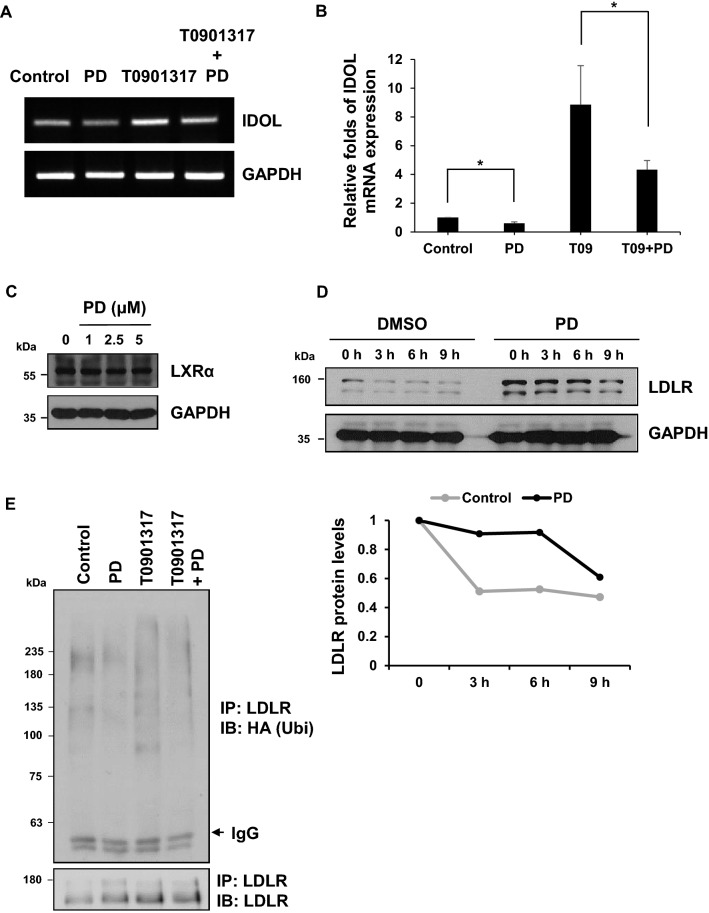
PD increases LDLR half-life by blocking LXR-induced *IDOL* expression. (**A**, **B**) HepG2 cells were treated with 10 μM T0901317 with or without 2.5 μM PD for 24 h. RT-PCR and Real-time PCR assay were performed to measure the mRNA levels of *IDOL* and *GAPDH* loading control. The *IDOL* mRNA expression levels represent the mean ± SD from three independent experiments. *P < 0.05 by one-way ANOVA with Tukey’s post hoc test. (**C**) HepG2 cells were treated with the indicated concentration of PD for 24 h. Western blot analysis was performed to determine the protein levels of LXRα and GAPDH from the cell lysates. (**D**) HepG2 cells were treated with either DMSO or 2.5 μM PD for 24 h and then added with 100 μg/mL CHX for the indicated time. LDLR protein was detected by western blotting and the intensity of LDLR protein was determined using Image J software and normalized to that of GAPDH control. (**E**) HepG2 cells were transfected with HA-ubiquitin plasmids (1 μg). The cells were treated with 2.5 μM PD or 10 μM T0901317 for 18 h and 50 nM Baf A1 for 6 h before being lysed. Cell lysates were immunoprecipitated with anti-LDLR and ubiquitinated LDLR was analyzed by immunoblotting as indicated.

Liver X receptors (LXRs) are cholesterol-sensing transcription factors that are activated in response to excessive intracellular cholesterol, inducing key genes involved in regulating cholesterol homeostasis including IDOL. Thus, LXR-IDOL pathway represents a mechanism for feedback inhibition of LDLR expression and cholesterol uptake. To investigate the effect of PD on the LXR-IDOL pathway, HepG2 cells were treated with T0901317, a synthetic LXR agonist in the presence or absence of PD and *IDOL* mRNA expression was examined. As shown in Fig. 5A,B, *IDOL* mRNA expression was increased by T0901317, but which was attenuated by the addition of PD. Meanwhile, there was no significant change in the protein levels of LXR α after PD treatment (Fig. 5C). These results indicate that PD inhibits the LXR-dependent *IDOL* expression, but not changed in levels of LXR protein.

Since IDOL promotes LDLR degradation through ubiquitination, the reduced expression of *IDOL* mRNA by PG and PD may contribute to enhance LDLR protein stability. To test this possibility, we chased LDLR protein levels after treatment of cycloheximide (CHX) which blocks new protein synthesis and found that the half-life of LDLR protein was increased in PD-treated cells compared to untreated cells (Fig. 5D). We next analyzed whether PD leads to changes in LDLR ubiquitination level by in vivo ubiquitination assay and found that, indeed, PD reduced the ubiquitination level of LDLR compared to untreated control. We further observed that PD can suppress the LDLR ubiquitination, which was enhanced by the activation of LXR-IDOL pathway upon T0901317 treatment (Fig. 5E). Taken together, these results indicate that PD enhances LDLR stability by inhibiting LXR-IDOL-mediated ubiquitination and degradation of LDLR.

### PG and PD exhibit a synergistic effect with simvastatin on hepatic LDLR expression and LDL-C uptake in HepG2 cells

**Figure 6:**
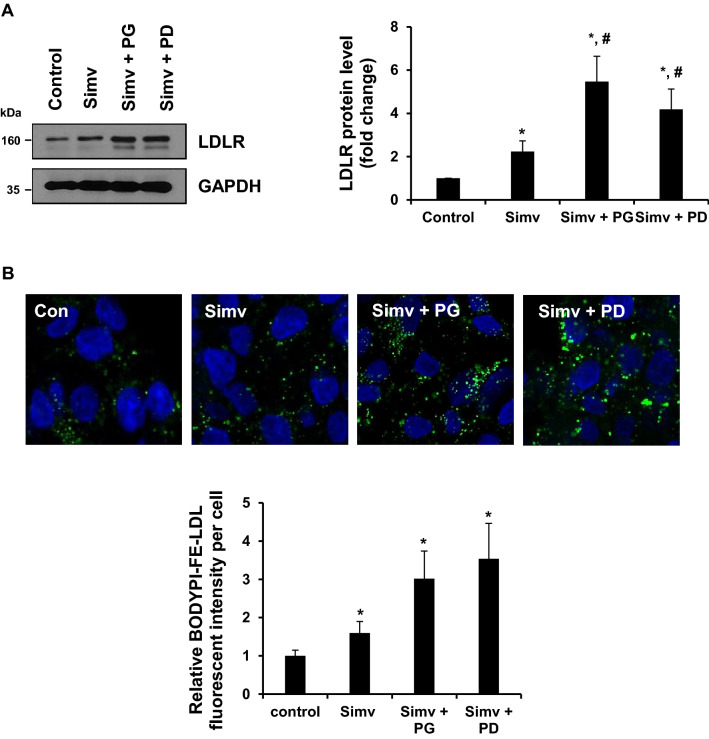
The synergistic effect of PG or PD with simvastatin to enhance LDLR expression and LDL-C uptake. (**A**) HepG2 cells were treated with 1 μM simvastatin with or without 250 μg/mL PG and 2.5 μM PD for 24 h. Cell lysates were subjected to western blotting with anti-LDLR and anti-GAPDH antibodies. The intensity of LDLR protein was measured using Image J software and normalized to that of GAPDH control. Bar graph shows the mean ± SD from three independent experiments. *P < 0.05 by one-way ANOVA with Tukey’s post hoc test. (**B**) HepG2 cells were treated with 1 μM simvastatin with or without 250 μg/mL PG and 2.5 μM PD for 24 h, followed by incubation with 5 μg/mL Bodipy FL dye-labeled LDL for 1 h and confocal microscopic imaging. Quantification of LDL fluorescence intensity per cells was analyzed using Image J software. DAPI (blue) was used for nuclear DNA staining. Bar graph represents the mean ± SD. *P < 0.05 by one-way ANOVA with Tukey’s post hoc test.

Statins are widely used for lowering blood levels of LDL-C, because it upregulates hepatic LDLR expression and enhances the subsequent uptake of LDL-C in the blood. To investigate whether PG and PD have a synergistic effect with statins on LDLR expression, we treated HepG2 cells with PG or PD together with simvastatin for 24 h and examined the expression levels of LDLR by western blot. Our results showed that simvastatin alone exhibits approximately 2.2-fold increase in hepatic LDLR level compared to untreated control cells. Importantly, the combined treatment with simvastatin and PG or PD synergistically increased LDLR levels to an average of 5.5 and 4.19-fold compared to control cells (Fig. 6A). In addition, a synergistic increase in LDL-C uptake was also examined upon co-treatment of PG or PD with simvastatin. As shown in Fig. 6B, LDL uptake assay showed that simvastatin alone promoted approximately 1.6-fold increase in the uptake of LDL particles compared to untreated cells. Meanwhile, PG or PD with simvastatin markedly increased the uptake of LDL particles around 3 and 3.5-fold, respectively, which were in accordance with the increased LDLR expression in Fig. 6A. These results suggest that PG and PD can be used in combination with statins for cholesterol-lowering therapy.

## Discussion

The link between high blood cholesterol and CVD have been well established, with the clearing of serum LDL-C by upregulation of hepatic LDLR being the therapeutic strategy. Previously, Zhao et al. have demonstrated that platycodin saponins from PG possess the anti-hypercholesterolemia activity on mice fed with high-fat diet^26,27^. In addition to these findings, the authors also revealed that PD is an active component in PG responsible for the cholesterol-lowering effect^22^. However, the molecular mechanisms underlying hypocholesterolemic action of PD have not been explored in the last decades. Here, we provide insight into the mechanism by which PD enhance LDL-C uptake in hepatic cells.

The changes of hepatic LDLR expression are driven by both transcriptional and post-translational regulation. Statins promote the transcriptional activation of hepatic *LDLR* gene by inhibiting the activity of HMG-CoA reductase, a rate-limiting enzyme in the pathway of cholesterol biosynthesis called mevalonate pathway, which leads to a decrease in intracellular cholesterol levels and subsequent activation of the SREBP-mediated gene expression. But, since the mevalonate pathway is not only essential for synthesis of cholesterol but also isoprenoid lipids including farnesyl diphosphate (FPP) and geranylgeranyl diphosphate (GGPP), used for protein prenylation process^28^, strategies aimed at blocking PCSK9-or IDOL-mediated post-translational modifications of LDLR have been considered as better approaches for LDLR upregulation^29^. While two anti-PCSK9 monoclonal antibodies, namely alirocumab and evolocumab, were approved for use to treat patients with familial hypercholesterolemia and CVD and ongoing clinical trial of the siRNA targeting PCSK9 named inclisiran yielded favorable outcomes^30^, drug development for anti-IDOL therapies is still at the preclinical stage.

In this study, we identified that PD contributed to upregulation of cell surface LDLR expression in HepG2 cells, importantly which was correlated with increased LDL-C uptake. In our attempt to search for the mechanism underlying the up-regulation of LDLR by PD, we found that PD prolonged the half-life of LDLR protein by down-regulating *IDOL* mRNA expression rather than promoting SREBP2-dependent induction of *LDLR* mRNA. Since the first identification of IDOL as a novel degrader of LDLR^31^, numerous studies have highlighted the important role of IDOL in LDL-C clearance. Genome-wide association studies identified genetic variations in *IDOL* locus that influence serum LDL-C levels^32–34^. Targeted gene sequencing of *IDOL* coding region demonstrated that individuals with loss-of-function *IDOL* variants were characterized by low circulating levels of LDL-C^35^. Moreover, results of *IDOL* gene disruption studies confirmed a role of IDOL in regulating LDL-C uptake through controlling LDLR abundance. Knockdown of *IDOL* with siRNA led to increased LDLR levels in HepG2 cells^13,36^. *IDOL*-null mouse embryonic stem cells displayed a marked increase in LDLR expression and LDL-C uptake independent of SREBP and PCSK9 pathway^37^. Thus, inhibiting the IDOL-mediated LDLR degradation pathway may offer a therapeutic benefit to enhance hepatic LDL-C clearance.

IDOL belongs to an E3 ubiquitin ligase to trigger specific degradation of LDLR^10^ and induced by LXRs that can be activated with LXR ligands such as oxysterols and synthetic agonists^37^. We also found that PD reduced the *IDOL* mRNA expression induced by synthetic LXR agonist T0901317, demonstrating that PD inhibits the LXR-dependent *IDOL* gene expression. The LXR-IDOL pathway can be inhibited by down-regulation of LXR expression^38,39^ or competitively inhibiting LXR agonist binding into the ligand-binding pocket of LXR protein^16^. Our data showed that LXRα expression was not changed by PD, suggesting that PD may antagonize LXR transcriptional activity without affecting LXR expression. The inhibitory effect of PD on LXR activity can be caused by blocking oxysterol binding to LXR ligand-binding pocket, reducing intracellular oxysterol concentration, or other mechanisms. Further studies are needed to uncover the details of molecular mechanism by which PD inhibits the LXR-IDOL pathway in HepG2 cells.

Is it possible that an effective concentration of PD is reached in the circulation after its dietary intake? A recent study determined that oral administration of PD at 20 mg/kg in rats resulted in the maximum plasma concentration (Cmax) of 44.45 ng/ml^15^***.*** Considering LDLR upregulation occur from 0.5 μM PD (Fig. 2A) which corresponds to 600 ng/ml, oral dosage of 270 mg/kg of PD is necessary to produce the biological effects. Because PD does not show any signs of toxicity against 14 principle organs upon oral administration of up to 2000 mg/kg in mice^40^, we can conclude that the serum concentration of PD required for effective hepatic LDLR elevation can be achieved after safe level of intake of PD.

Through more than 25 years of clinical trials, statins were established as the first line treatment for lowering LDL-C levels in CVD patients^41^. When stain monotherapy is not sufficient for achieving treatment goal for LDL-C, the addition of other cholesterol-lowering drugs to statins may be considered. The statin combination therapy also allows for reducing statin doses for patients with statin intolerance or other side effects. Several clinical studies have demonstrated that the combination of statins and ezetimibe, an intestinal cholesterol absorption inhibitor, provided further mean reduction of LDL-C by 15–30% when compared with statin monotherapy^42–45^. Furthermore, the addition of PCSK9 inhibitors such as alirocumab and evolocumab on a background of statin therapy induced a significant decrease in LDL-C levels and reduced several cardiovascular risk factors^46,47^. In this respect, several natural compounds have been tested for their synergistic effects with statins. The results showed that PCSK9 inhibitors such as curcumin, epigallocatechin gallate, and tanshinone IIA, and IDOL inhibitor such as xanthohumol enhanced the statin-mediated induction of *LDLR* mRNA and LDL-C uptake^16,48–50^. In the present study, we demonstrated that co-treatment of HepG2 cells with PD and simvastatin resulted in further increase in LDLR expression and LDL-C uptake compared to simvastatin alone. What is the molecular mechanism for the synergistic effect of PD with statins on LDLR upregulation? Statins activate LDLR gene transcription and PD increases LDLR protein stability. Therefore, the combined use of these drugs with different mechanisms of action might show synergistic effects to elevate LDLR levels via prolonging LDLR protein half-life that is transcriptionally induced upon statins. Additionally, because stains were also known to suppress IDOL expression^13^, treatment of both drugs may lead to cumulative effects on IDOL downregulation.

**Figure 7:**
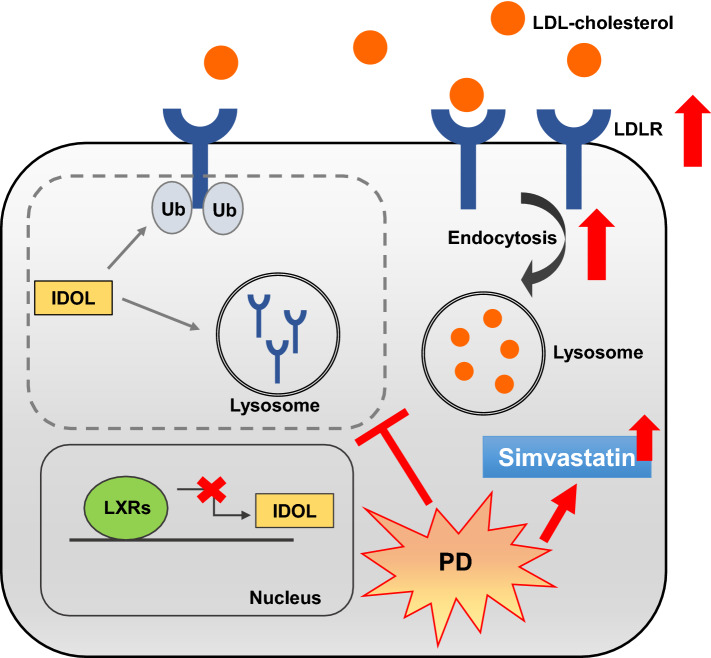
Schematic model for the hypocholesterolemica action of PD. PD enhances LDL-C uptake via increasing hepatic LDLR protein stability through inhibiting LXR-IDOL pathway. Furthermore, co-treatment of PD with simvastatin enhances their cholesterol-lowering effect.

In conclusion, we demonstrated that PD induces LDLR expression and uptake of LDL-C particles by inhibiting the LXR-IDOL pathway. In addition, we showed that PD has synergistic effects with statin for upregulation of LDLR expression and uptake of LDL-C (Fig. 7), suggesting that PD, with its potent inhibitory activity towards IDOL, could be useful as an adjunctive therapy to statins.

## Supplementary information

Supplementary Information

## Data Availability

All data and materials are described within the article.
