# Supplementary material for: Platycodin D enhances LDLR expression and LDL uptake via down-regulation of IDOL mRNA in hepatic cells
Source: Sci Rep. 2020 Nov 16;10:19834. doi: 10.1038/s41598-020-76224-w (PMC7670405; doi:10.1038/s41598-020-76224-w)
Supplement: Supplementary file 1 — Supplementary Information [file 41598_2020_76224_MOESM1_ESM.docx]

**Platycodin D enhances LDLR expression and LDL uptake via down-regulation of *IDOL* mRNA in Hepatic cells**

Yu-Jeong Choi,^1^ Sol Ji Lee,^1,2^ Hyo In Kim,^1^ Hee Jung Lee,^3^ So Jung Kang,^4^ Tai Young Kim,^2,5,*^ Chunhoo Cheon,^5^ and Seong-Gyu Ko^5,*^

^1^ Department of Science in Korean Medicine, Graduate School, Kyung Hee University, Seoul, 02447, Republic of Korea

^2^ Center for Cognition and Sociality, Institute for Basic Science, Daejeon, 34126, Republic of Korea

^3^Department Global Public Health and Korean Medicine Management, College of Korean Medicine, Graduate School, Kyung Hee University, Seoul, Republic of Korea

^4^ Department of Clinical Koeran Medicine, Graduate School, Kyung Hee University, Seoul, Republic of Korea

^5^ Department of Preventive Medicine, College of Korean Medicine, Kyung Hee University, Seoul, 02447, Republic of Korea

*To whom correspondence should be addressed:

Dr. Tai Young Kim

Center for Cognition and Sociality, Institute for Basic Science, Daejeon, 34126, Republic of Korea

Email: [taik@ibs.re.kr](mailto:taik@ibs.re.kr), Fax: 82-42-878-9151, Phone: 82-42-878-9155

Dr. Seong-Gyu Ko

Department of Preventive Medicine, College of Korean Medicine, Kyung Hee University, 1 Hoegi, Seoul, 130-701, Korea

Email: [epiko@khu.ac.kr](mailto:epiko@khu.ac.kr), Fax: 82-2-966-1165, Phone: 82-2-961-0329

**Supplementary figures and figure legends**


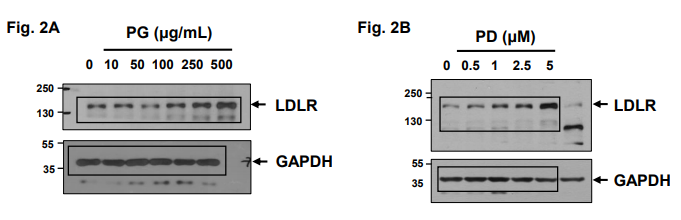


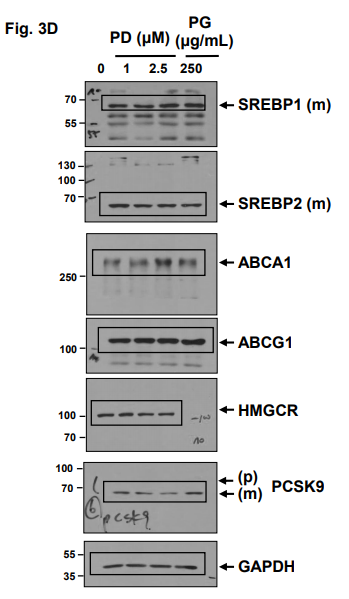

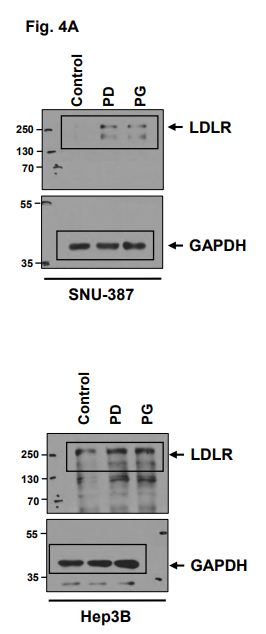


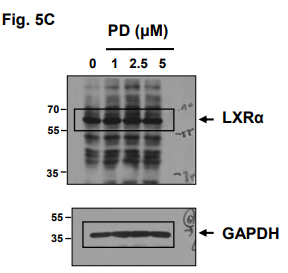

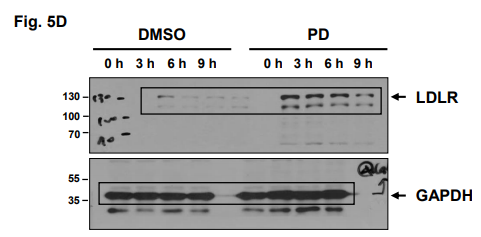


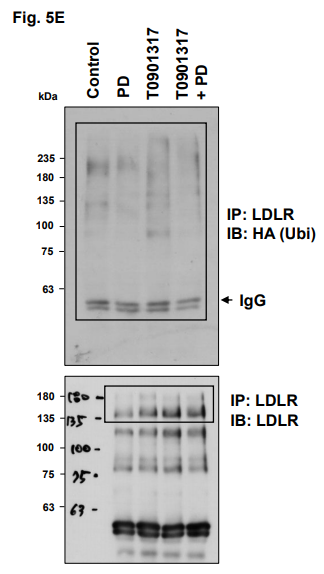


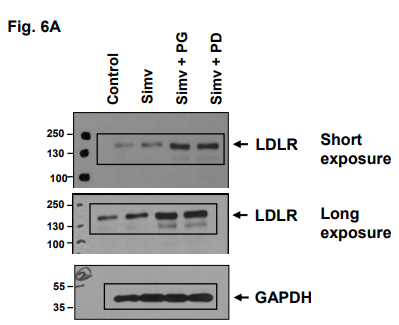


Supplementary Figure S1. Uncropped western blot images.


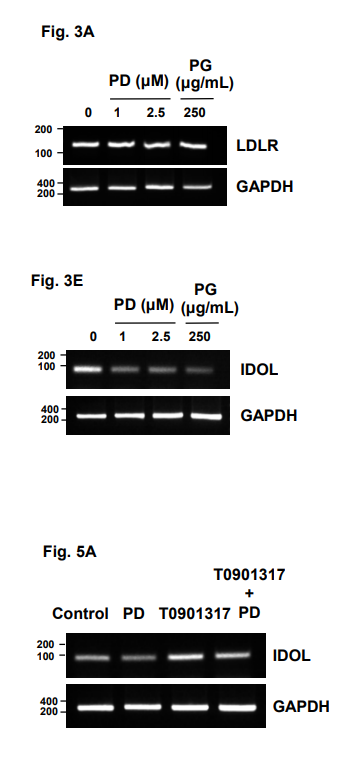


Supplementary Figure S2. Uncropped PCR images.
